# Brevilin A, a Novel Natural Product, Inhibits Janus Kinase Activity and Blocks STAT3 Signaling in Cancer Cells

**DOI:** 10.1371/journal.pone.0063697

**Published:** 2013-05-21

**Authors:** Xing Chen, Yuping Du, Jing Nan, Xinxin Zhang, Xiaodong Qin, Yuxin Wang, Jianwen Hou, Qin Wang, Jinbo Yang

**Affiliations:** 1 School of Life Sciences, Lanzhou University, Lanzhou, Gansu, People’s Republic of China; 2 Department of Molecular Genetics, Lerner Research Institute, The Cleveland Clinic, Cleveland, Ohio, United States of America; Case Western Reserve University, United States of America

## Abstract

Signal abnormalities in human cells usually cause unexpected consequences for individual health. We focus on these kinds of events involved in JAK-STAT signal pathways, especially the ones triggered by aberrant activated STAT3, an oncoprotein which participates in essential processes of cell survival, growth and proliferation in many types of tumors, as well as immune diseases. By establishing a STAT3 signal based high-throughput drug screening system in human lung cancer A549 cells, we have screened a library from natural products which contained purified compounds from medicinal herbs. One compound, named Brevilin A, exhibited both strong STAT3 signal inhibition and STAT3 signal dependent cell growth inhibition. Further investigations revealed that Brevilin A not only inhibits STAT3 signaling but also STAT1 signaling for cytokines induced phosphorylation of STAT3 and STAT1 as well as the expression of their target genes. In addition, we found Brevilin A could attenuate the JAKs activity by blocking the JAKs tyrosine kinase domain JH1. The levels of cytokine induced phosphorylation of STATs and other substrates were dramatically reduced by treatment of Brevilin A. The roles of Brevilin A targeting on JAKs activity indicate that Brevilin A may not only be used as a STAT3 inhibitor but also a compound blocking other JAK-STAT hyperactivation. Thus, these findings provided a strong impetus for the development of selective JAK-STAT inhibitors and therapeutic drugs in order to improve survival of patients with hyperactivated JAKs and STATs.

## Introduction

The outline of JAK-STAT signal pathway has been finished nearly 20 years ago [1]. More studies were then continued for signal details including protein interactions, post-modifications, transcriptional regulations, and physiological effects. The Janus kinase (JAK) family contains four tyrosine kinase members, including JAK1, JAK2, JAK3 and Tyk2, which transduce cytokine-induced signals via Signal Transducers and Activators of Transcription (STATs). Usually, receptor associated JAKs were activated upon receptor dimerization in the presence of cytokines. Meanwhile STATs in the cytoplasm were recruited to the receptors and phosphorylated by JAKs. Tyrosine phosphorylated STATs formed homo- or heterodimers through phosphotyrosine-SH2 interactions, and translocated into the nucleus to initiate transcriptions of targeted genes [2]. Abnormal activity of JAK-STAT signals has been considered to be link to many diseases, including cancers and immune disorders. Aberrated STATs activity usually correlates with various types of tumor growth, and progression of diverse cancer malignancies, both in response to cytokines and by mutant protein tyrosine kinases. Of the seven STAT family members (STAT1-STAT6, with two independent genes encoded STAT5A and STAT5B), STAT3, as well as STAT5 to some extent, are most frequently activated in quite a lot human solid tumors and leukemias [3]–[5].

In many STAT3 constitutive activated cancer cells, either cultured human tumor cells or generated mouse models, blocking STAT3 signaling will inhibit cell growth, induce apoptosis and reduce cell metastasis. In glioma or glioblastoma cells [6], [7], breast carcinoma cells [8], colon cancers [9], squamous cell derived tumors [10], prostate cancer cells [11]–[13] and melanomas [14], [15], targeting disruption of STAT3 activity by interfering RNAs, expressing dominant negative STAT3 forms or applying specific signaling inhibitors would remarkably down regulate STAT3 induced genes, including CyclinD1, Bcl-xl, c-Myc, Survivin and other genes regulating cell cycles and cell proliferation, and then subsequently reduce cell growth and enhance cell apoptosis [16], [17]. Metastasis is the main cause of poor prognosis and caner-related deaths compared with tumor genesis and neoplasm growth. STAT3 now has been considered as one of the critical oncoproteins mediating regulation of cell invasion and tumor microenvironment. In human colorectal cancers, STAT3 was activated in those who got poor prognosis [18]. Proteins involved in migration and invasion of cancer cells, like matrix metalloproteinases (MMP-1, MMP-2, MMP-10, *etc*.) and Twist, were regulated by STAT3 activation [19]–[21]. An IL-6 induced JAK/STAT3 signaling was essential for infiltration of circulating cancer cells. Tumor-derived IL-6 helps circulating breast carcinoma and melanoma to re-establish *in situ* or at distant metastasis regions [22]. Recently, it has been reported that persistently activated STAT3 maintained NF-κB activity through p300 mediated pathways. NF-κB activity dramatically decreased by STAT3 RNAi in many STAT3 constitutive activated cancer cells [23], suggesting that STAT3 inhibitors may also play potential roles in blocking NF-κB activity and enhancing growth inhibition in these cancer cells.

Exploring JAK-STAT signal inhibitors especially STAT3 inhibitors by high throughput drug screening (HTS) is an efficient way in discovering potential specific drugs targeting on STAT3 or upstream JAK kinases. *My N. Chau* and colleagues developed a prostate cancer cell line which contained a STAT3 reporter construct for high throughput screening of STAT3 activators and inhibitors [24]. Here we established a similar STAT3 signaling based luciferase reporter screening system in a human lung cancer cell line A549, which shows constitutive activated STAT3 activity and could be further induced by cytokines like IL-6, EGF, and HGF [25]. By screening, Brevilin A, a novel natural product, showed significant JAK-STAT signaling inhibition without immediate-direct cell toxicity from 1,400 more compounds which were originally isolated from plants, most of which were known as herbal remedies. Brevilin A has preferred cell growth inhibition of DU145 and MDA-MB-468, those growths are dependent on STAT3 signaling [17], [26]. Further investigation revealed that Brevilin A blocked activity of Janus Kinase Tyrosine Kinase JH1 domain, and then reduced phosphorylation of downstream effectors. Brevilin A may act as a potential drug targeting on diseases caused by JAK-STAT abnormalities.

## Materials and Methods

### Antibodies and Reagents

Antibodies against STAT3, JAK2, pTyr705-STAT3, pTyr701-STAT1, pSer473-AKT, pSer9-GSK-3β, c-Myc, CyclinD1, PARP, pTyr1007/1008-JAK2, pTyr1054/1055-TYK2, pSer536-p65 and p65 were obtained from Cell Signaling Technology; Antibodies against c-Src, pTyr (PY99), GAPDH and His-tag were obtained from Santa Cruz Biotechnology, Inc.; pGL4.20 vector and luciferase substrate Steady Glo were obtained from Promega; M-MLV first strand cDNA synthesis kit were obtained from Invitrogen, Life Technologies Corporation; PD180970, AG490, Staurosporine, Doxorubicin, ATP and EZview Red ANTI-FLAG M2 Affinity beads were purchased from Sigma-Aldrich; Interleukin-6(IL-6), Interferon α (IFNα) and Interferon γ (IFNγ) was from PeproTech. Ni^+^ affinity chromatography beads were obtained from GE Healthcare Life Sciences. 10× PK kinase buffer were obtained from New England Biolabs (NEB).

### Plasmids and Cell Lines

A sequence containing 16× SIE plus with one TATA box was inserted into pGL4.20 between KpnI and HindIII. The SIE-luc-puro construct was transfected into A549 cell line. Forty-eight hours after transfection, cells were selected with 5 µg/ml puromycin for 2 weeks, then 2.5 µg/ml for another 2 weeks. Clones were picked up and analyzed separately. Sequences encoding human JAK1-JH1 domain, JAK2-JH1 domain, JAK3-JH1 domain, Tyk2-JH1 domain and c-Src were cloned into plv-SV40-puro lentivirus expression vector separately. Additional sequences of Flag-His dual tags were fused at the C-terminal of each JAKs-JH1 domain. c-Src were fused with single Flag tag at the C-terminal. Each of above constructs was transfected into HEK293T combined with pMD-2.G and pCMV-dr8.74 helper vectors for virus packaging. Supernatant media was collected after 48 h and used to infect HEK293T overnight, then replaced with fresh media for another 24 h. Stable cell pools were selected in the presence of puromycin (2.5 µg/mL) for 7 days.

### Cell Culture

Cells were cultured in Dulbecco's modified Eagle medium supplemented with 10% fetal bovine serum (FBS), penicillin (100 U/ml) and streptomycin (100 µg/ml).

### Drug Screening

Natural products for drug screening were from National Compound Resource Center (The Original library supplier for this institute was BioBioPha Co., Ltd.). Compounds from natural products (10 mM) were diluted with DMEM (10% FBS) to 100 µM (Diluted Compounds). A549R cells for drug screening were plated in 96-well plates at a density of 1×10^4^ (100 µl/well in DMEM with 10% FBS). Twelve hours later, 25 µl Diluted Compounds with 75 µl fresh DMEM (10% FBS) were added into each separated well for another 24 h for the 1^st^ round screening at the concentration of 25 µM. 12.5 µl Diluted Compounds with 87.5 µl fresh DMEM were added for the 2^nd^ round screening at the concentration of 12.5 µM. DMSO was used as vehicle (0.25% in 1^st^ round screening and 0.125% in 2^nd^ round screening). IL-6 (250 ng/ml) and PD-180970 (250 nM) were used as known stimulator and inhibitor to check system response for each round of screening in a single plate. The system response would be considered normal when IL-6 induces more than 2.5 fold fluorescence and PD-180970 shows 40%–50% fluorescence inhibition in each round screening. We used a counterscreen by assuming that the known inhibitor PD-180970 has significant signal inhibition, and potential inhibitors would always have better performances than PD-180970. Since the positive control PD-180970 (250 nM) always showed a fluorescence ratio approximate at 50% and could inhibit STAT3 phosphorylation significantly when judged by Western-Blot analysis, we chose 50% as a “cut off” value, then any compound that exhibits a fluorescence ratio of control cells ≤50% (*i.e.* Fluorescence inhibition ≥50%) will be picked out. The details are summarized as follows: Step 1, 1^st^ round screening, One well-One compound, 25 µM, luciferase assay only. Compounds were picked out whenever FR (Fluorescence Ratio) is ≤50%. After this step, the picked compounds might include some overly toxic ones. To rule out fluorescence inhibition caused by cytotoxicity, Step2 was applied. Step 2, 2^nd^ round screening, 12.5 µM of each compound from Step 1, and two repeats for luciferase and MTT assays were applied. If FR% is ≤50% & Δ(CV% – FR%) is ≥30%, the compounds will be picked out for further analyses. The overly toxic compounds were excluded by this step. The deviation “30%” is an empiric value that was able to distinguish overly toxic compounds and specific compounds. Here, FR, Fluorescence Ratio = Fluorescence value of treated well divided by Fluorescence value of control well; CV, Cell Viability = Cell survival value of treated well divided by Cell survival value of control well; Luciferase assay was performed for Fluorescence Value; MTT assay was performed for Cell Survival Value. (Percentage of Fluorescence inhibition = 100% – FR%; Percentage of Cell growth inhibition = 100% – CV%).

For the luciferase assay, 50 µl luciferase substrate Steady Glo were added (50 µl/well). After 10 minutes incubation, fluorescence was measured by Vector3 Multilevel Plate Counter (Perkin Elmer). For the MTT cell viability assay, 20 µl MTT solution (5 mg/ml in PBS) was added for 4 hours incubation. The resultant crystals were dissolved in 100 µl DMSO and the absorbance intensity was measured by Vector3 at 490 nm wavelength.

### Western-Blot, Immunoprecipitation and Cell Staining

Cells were washed with ice-cold PBS for three times and lysed with RIPA lysis buffer for 30 minutes at 4°C (150 mM NaCl, 1% NP-40, 0.5% Sodium Deoxycholate, 0.1% SDS, 50 mM Tris, pH 7.5, 5 mM EDTA, 1 mM EGTA, 1× protease inhibitor cocktail (Roche), 1× phosphatase inhibitor cocktail (Roche)). The lysates were centrifuged at 12,000× rpm for 10 minutes at 4°C. Equal quantities of proteins, determined by BCA method (Pierce, Thermo), were then separated by SDS-PAGE and transferred to PVDF membranes (Millipore, Merck). Proteins were detected with indicated antibodies.

HEK293T cells expressing Flag tagged Src were pretreated with DMSO, PD180970 (500 nM) and Brevilin A (15 µM) for 4 hours separately. Cells were washed with ice-cold PBS for three times and lysed with 500 µl lysis buffer (50 mM Tris HCl, 150 mM NaCl, 1 mM EDTA, 1% Triton X-100 pH 7.4) in the presence of protease inhibitor cocktail and phosphatase inhibitor cocktail. The lysates were centrifuged at 12,000× rpm for 10 minutes at 4°C. Equal quantities of proteins by BCA methods, were then incubated with ANTI-FLAG M2 Affinity beads for 8 hours at 4°C. Src protein samples were eluted with 0.1 M Glycine-HCl, pH 3.5 and neutralized with Tris-HCl (0.5 M pH 7.4).

For apoptosis assay, cells were plated in 24-well plates. Twelve hours later, media was removed and replaced with fresh media in the presence of 10 µM Brevilin A for 24 h. Cells were then subjected to an Annexin-V-PI dual staining process as in the protocol of Annexin V-FITC Apoptosis Detection Kit (Beyotime Institute of Biotechnology).

### Protein Purification and Kinase Assay

C-terminal His-tagged hSTAT3 recombinant protein was expressed in *E.coli*, *Rosetta* and purified by Ni^+^ affinity chromatography. *hstat3* CDS was cloned into pET28b, and induced by 0.5 mM isopropylthio-β-galactoside at 37°C for 6 h. Inclusion bodies were centrifuged at 12,000× rpm for 10 minutes at 4°C after ultrasonication treatment on whole *E.coli* cells. Then the inclusion bodies were lysed with lysis buffer (0.5 M NaCl, 20 mM sodium phosphate, 20 mM imidazole, 8 M urea, pH 7.5). Ni^+^ affinity chromatography beads were then used for unfolded His-tagged hSTAT3 binding. On-column Refolding was chosen and finally the refolded STAT3 protein was eluted by elution buffer (0.5 M NaCl, 20 mM sodium phosphate, 250 mM imidazole, pH 7.5). After an ion exchange process, the purified hSTAT3 protein in PBS (10% glycerol) was frozen for further analysis.

Approximate 5×10^8^ HEK293T cells expressing Flag-His tagged Tyk2-JH1 were harvested and lysed with lysis buffer (0.5 M NaCl, 20 mM sodium phosphate, 20 mM imidazole, pH 7.5). Ni^+^ affinity chromatography beads were then used for Flag-His-tagged Tyk2-JH1 binding. Protein was eluted with 250 mM imidazole and diluted with ANTI-FLAG M2 Affinity beads binding buffer and incubated with M2 Affinity beads for 2 h at room temperature. Tyk2-JH1 protein was finally eluted with PBS containing 3× FLAG peptide (500 ng/ml, 30 min at room temperature) for further kinase assay.

Approximate 150 ng hSTAT3 protein and 20 ng Tyk2-JH1 kinase were pre-incubated with 1× kinase buffer, in the presence of concentration series at 10, 20, 40, and 80 µM, for 10 min. ATP was added into the reaction at the concentration of 200 µM to 50 µl finally volume. The kinase reaction was then continued at 37°C for 2 h, and it was stopped by 5× protein sample loading buffer (95°C, 5 min). 20 µl of each sample was loaded for SDS-PAGE and Western-Blot analysis.

### RT-PCR and Quantitative Real-time PCR

Total mRNA was extracted from cultured cells with TianGen DNA purification kit. Reverse transcription was performed with M-MLV reverse transcription kit (Invitrogen, Life Technologies Corporation). Quantitative real-time PCR was finished with Roche Cyber Green PCR mix kit on Biorad C1000 Thermal Cycler. The primer pairs for RT-PCR were as follows: *gapdh* forward 5′-TGGCAAATTCCATGGCAC-3′, reverse 5′-CCATGGTGGTGAAGACGC-3′; *socs3* forward 5′-CCATGGTGGTGAAGACGC-3′, reverse 5′-CCTGTCCAGCCCAATACCTGA-3′ [27]; *IRF-1* forward5′-CGATACAAAGCAGGGGAAAA-3′, reverse 5′-TAGCTGCTGTGGTCATCAGG-3′ [28].

### Data Analysis and Statistical Methods

Each analysis was repeated as denoted. Relative cell viability was expressed as a percentage (%) relative to the untreated control cells. Error bars represented standard deviation (±SD). Data was analyzed by ANOVA method for each two-group comparison tests. Blot and image signal intensity was quantified using ImageJ2X software. P-STAT3 and p-p65 fold changes were normalized to total STAT3 and p65 respectively, while p-AKT and p-GSK-3β changes were normalized to GAPDH.*socs3* and *IRF-1* mRNA level changes were normalized to total *gapdh* mRNA. Quantification numbers are represented in the bottom of the blots. Fold changes of Annexin-V fluorescence were normalized by cell counting. IC_50_ (GI_50_) was calculated by SPSS19 software (regression-probit method). Histograms and diagrams were drawn with Origin 8 software.

## Results

### 

#### Establishment of STAT3 signaling based high-throughput drug screening system

**Figure 1:**
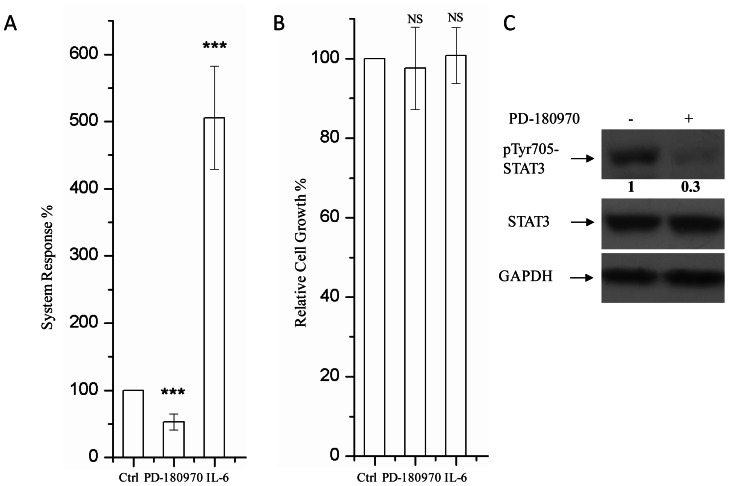
Stable cell line A549R responses to both IL-6 and PD-180970. Luciferase (A) and MTT (B) assays of A549R cells treated with PD-180970 (250 nM) and IL-6 (250 ng/ml) respectively. A549R cells were plated in 96-well plates at a density of 1×10^4^ (100 µl/well in DMEM with 10% FBS). Twelve hours later, media were removed and replaced with fresh media in the presence of IL-6 or PD-180970. Bars show the standard deviation (±SD) (three independent repeats, n = 5 in each repeat). ***p<0.001, **p<0.01,*p<0.05, significant relative to vehicle control. NS, no statistical significance. (C) PD-180970 inhibits STAT3 activity in A549R cells. A549R cells were plated and cultured in 100-mm dishes at 70% confluence. Then cells were treated with PD-180970 (250 nM) for 24 h. DMSO was used as control.

A549 cells were transfected with luciferase reporter vector, SIE-luc-puro, which contains repeated STAT3 response elements (16× SIE, sis-inducible element,5′-TTTCCCGTAAAATTCCTGTAAGTTTCCCGTAAAATTCCTGTAAGTTTCCCGTAAAA TTCCTGTAAGTTTCCCGTAAAATTCCTGTAAGTTTCCCGTAAAGCTCGCTAGCATTCCTGTAA GTTTCCCGTAAATTCCTGTAAGT TTCCCGTAAATTCCTGTAAGTTTCCCGTAAATTCCTGTAA GCTCGAGGATATCAAGATCTAGACACTAGAGGGTATATAATGGAAGCTCGACTTCCAGCTT-3′) (Fig. S1). Stable cell line A549R from a single clone was chosen then. This clone was able to response to both cytokines and inhibitors involved in STAT3 signaling (Fig. 1A). IL-6 induced approximately 5× fold fluorescence, and PD-180970 treatment showed about 50% inhibition of luciferase activity. The concentrations of IL-6 and PD-180970 for treatments didn’t affected cell growth significantly (Fig. 1B). PD180970, the known Src kinase inhibitor, was able to inhibit STAT3 activity partly in A549 cell line as reported [25] (Fig. 1C).

### Identification of Brevilin A as a STAT3 Signaling Inhibitor

**Figure 2:**
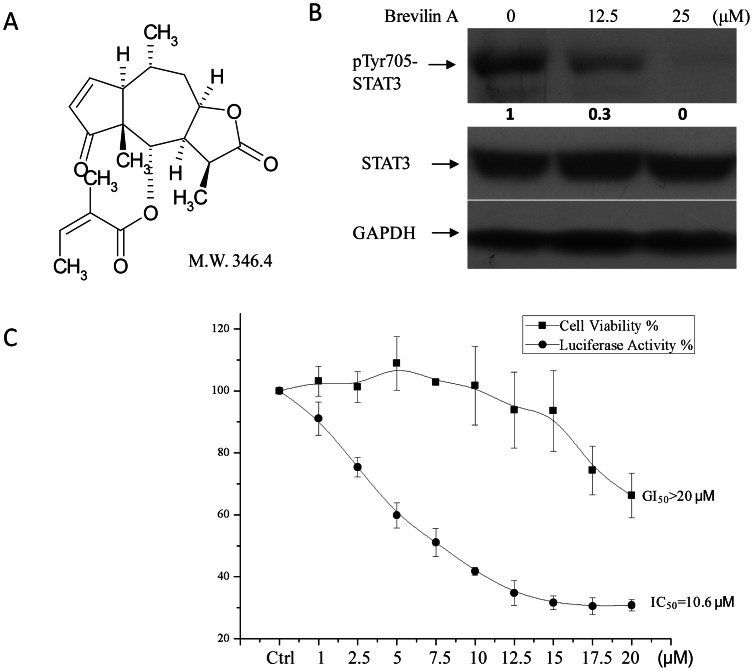
Brevilin A inhibits STAT3 signaling in a dose dependent manner. (A) Structure of Brevilin A. (B) Brevilin A inhibits STAT3 phosphorylation in A549R cells. A549R cells were plated and cultured in 100 mm dishes at 70% confluence. Cells were then treated with Brevilin A (12.5 µM and 25 µM ) for 24 h. DMSO was used as control. (C) STAT3 signaling was inhibited by Brevilin A in a dose dependent manner. A549R cells were plated in 96-well plates at a density of 1×10^4^ (100 µl/well in DMEM with 10% FBS). Twelve hours later, media was removed and replaced with fresh media in the presence of Brevilin A at different concentrations for another 24 h (20 µM, 17.5 µM, 15 µM, 12.5 µM, 10 µM, 7.5 µM, 5 µM, 2.5 µM and 1 µM). Luciferase and MTT assays were performed then. Bars show the standard deviation (±SD) (3 independent repeats, n = 5 in each repeat).

Compounds (1,440 in total) from natural products (Table S1) were screened as described in *Materials and Methods*. In the 1^st^ round screening, also considered as a rough screening, one compound-one well strategy at the concentration of 25 µM was used. Nine compounds showed more than 50% fluorescence inhibition (Table S2, values in red). In the 2^nd^ round screening, 12.5 µM compounds were chosen for further luciferase assay, as well as for additional MTT cell viability assay. Only one compound, named Brevilin A (Fig. 2A, a pseudoguaiane sesquiterpene from *Litsea glutinosa*, information provided by the original supplier) still showed more than 50% fluorescence inhibition, while exhibited a deviation (more than 30%) between cell viability (CV) and fluorescence ratio (FR). We speculate that signal specific inhibitors should exhibit more signal inhibition than cell growth inhibition within 24 hours, and in the 2^nd^ round screening, if FR% is ≤50% andΔ(CV% - FR%) is ≥30%, the compounds will be picked out for further analyses (Table S3). Of the 9 compounds from 1^st^ round screening, only Brevilin A met these criteria (Fig. S2). It seemed that we could get same results by evaluating Z scores in the 1^st^ round screening (Table S4). Western-Blot further proved that Brevilin A blocked STAT3 tyrosine 705 phosphorylation at the concentration of referred 12.5 and 25 µM for 24 h treatment in A549R cells (Fig. 2B). Signal inhibition and cell viability were then analyzed by luciferase and MTT assay at serial concentrations of Brevilin A treatment after 24 h (Fig. 2C). Brevilin A exhibited better STAT3 signaling inhibition in a dose dependent manner (IC_50_ = 10.6 µM) than cell viability inhibition within 24 h (GI_50_>20 µM), indicating that it’s a signal specific inhibitor more than a compound that directly kills cultured cells based on cell toxicity. We then chose concentrations around 10 µM for further analyses.

### Brevilin A Inhibits Constitutively Activated-STAT3 Driven DU145 and MDA-MB-468 Cells

**Figure 3:**
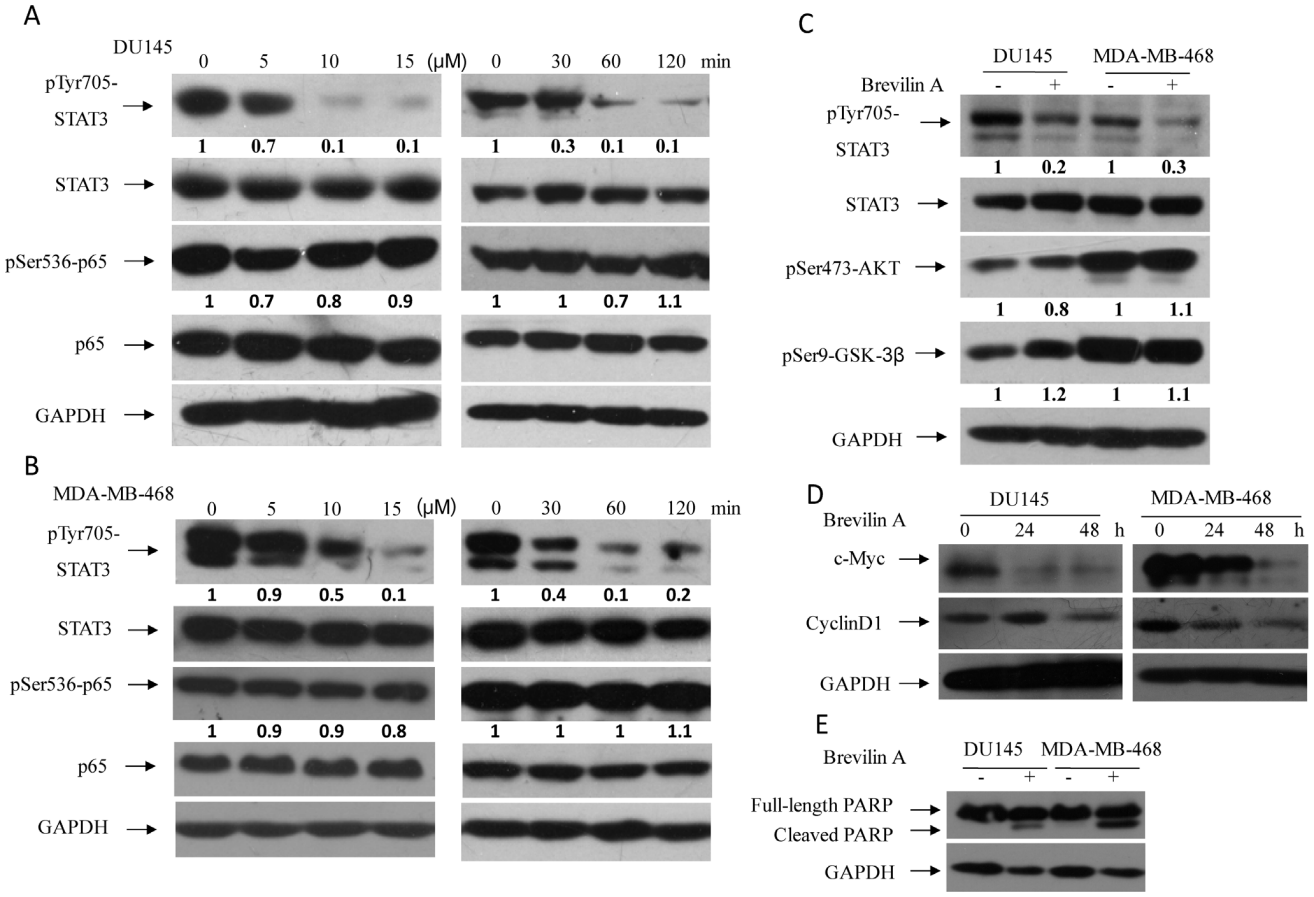
Brevilin A inhibits constitutive activated STAT3 in DU145 and MDA-MB-468 cells. STAT3 tyrosine 705 phosphorylation was inhibited by Brevilin A in dose (5 µM, 10 µM and 15 µM for 2 h) and time (10 µM for 30 min, 60 min and 120 min) dependent manners in both DU145 (A) and MDA-MB-468 (B) cells, while phosphorylation of p65-Ser536 (A and B), AKT-Ser473 and GSK-3β-Ser9 (C) was not affected correspondingly (10 µM, 2 h). (D) c-Myc and CyclinD1 decreased after 24 h and 48 h treatment with Brevilin A (10 µM). Cells were plated and cultured in 100 mm dishes for 12 h, then were treated with Brevilin A as described. (E) Cleaved PARP increased in DU145 and MDA-MB-468 cells with Brevilin A (10 µM) treatment for 24 h. DMSO was used as control.

**Figure 4:**
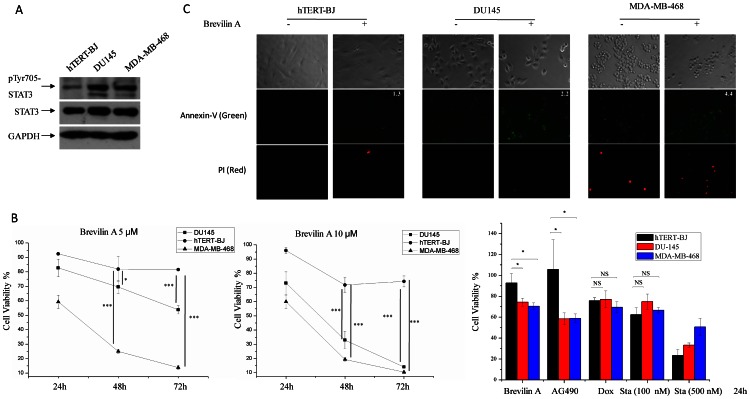
Brevilin A selectively inhibits cell growth of DU145 and MDA-MB-468 cells. (A) STAT3 tyrosine 705 phosphorylation was detected in hTERT-BJ, DU145 and MDA-MB-468 cells. (B) Left and middle diagrams, hTERT-BJ, DU145 and MDA-MB-468 cells were plated in 96-well plates at a density of 8×10^3^ (100 µl/well in DMEM with 10% FBS). Twelve hours later, media were removed and replaced with fresh media in the presence of Brevilin A (5 µM and 10 µM) for 24 h, 48 h and 72 h, cell viability was measured by MTT assay. Right histogram, 12 hours later cells were plated, media were removed and replaced with fresh media in the presence of Brevilin A (10 µM), AG490 (100 µM), Doxorubicin (1 µM) and Staurosporine (100 nM and 500 nM) for 24 h. Dox, Doxorubicin. Sta, Staurosporine. Bars show the standard deviation (±SD) (3 independent repeats, n = 3 in each repeat). ***p<0.001, **p<0.01, *p<0.05. NS, no statistical significance. (C) Cells were plated in 24-well plates. Twelve hours later, media was removed and replaced with fresh media in the presence of 10 µM Brevilin A for 24 h. DMSO was used as control. Cells were then subjected to an Annexin-V-PI dual staining process. Same exposure program was used at each wavelength.

Human prostatic carcinoma DU145 and breast cancer MDA-MB-468 cell lines showed constitutive STAT3 activity. Then we ask whether Brevilin A could inhibit STAT3 activity in these two cell lines. Figure 3A and B indicated that Brevilin A inhibits STAT3 signaling in dose- and time-dependent manner in both DU145 (Fig. 3A) and MDA-MB-468 (Fig. 3B). To test signal specific inhibition, levels of phosphorylation of p65-Ser536, AKT-Ser473 and GSK-3β-Ser9 were analyzed. Interestingly, Brevilin A did not exhibit corresponding effects on phosphorylation of these proteins (Fig. 3A, 3B and 3C), indicating that Brevilin A may not affect or has less effects on other cell signals. Inhibition of STAT3 activity usually leads to down-regulation of target genes, *e.g.*, c-Myc and CyclinD1 [17]. Here, after treated with Brevilin A for 24 h and 48 h, both c-Myc and CyclinD1 expression reduced in DU145 and MDA-MB-468 cells (Fig. 3D). Increased cleaved PARP was also observed (Fig. 3E), indicating that Brevilin A induced DU145 and MDA-MB-468 apoptosis after 24 h treatment [29]. It is consistent with the reports that blocking STAT3 activity led to cell growth inhibition in DU145 [30] and MDA-MB-468 cells [31]. Then cell viability was measured for DU145 and MDA-MB-468 cells, as well as human non-transformed telomerase-immortalized fibroblasts BJ cells (hTERT-BJ, a immortalized normal cell line). hTERT-BJ cells had lower STAT3 activity (Fig. 4A) and thus were used as negative control cells. After treated with Brevilin A for 24 h, 48 h and 72 h, Brevilin A showed more significant cell growth inhibition on DU145 and MDA-MB-468 than hTERT-BJ at both 5 µM and 10 µM concentration (Fig. 4B, left and middle). Several other compounds, the mechanisms of which were known on cell viability, were chosen as controls. AG490, a JAK inhibitor, could inhibit JAK-STAT signaling dependent cell growth (*e.g.*, DU145 and MDA-MB-468); Staurosporine, which is a known pan-tyrosine kinase inhibitor [32], inhibits lots of cell processes and usually shows no cell type specificity; Doxorubicin, a wildly used compound, is able to induce cell apoptosis and block cell growth [33]. By comparing the effects on cell viability among DU145, MDA-MB-468 and hTERT-BJ cells after 24 hours drug treatment (Fig. 4B, the right histogram), AG490 shows similar effects (with Brevilin A) on these cells, while Doxorubicin and Staurosporine had no specificity on cell viability or growth among these cells. Further investigation by Annexin-V staining revealed that Brevilin A exhibited a stronger induction of apoptosis for DU145 and MDA-MB-468 (about 2.2 and 4.4 fold, respectively) than hTERT-BJ (∼1.3 fold) after 24 h treatment (Fig. 4C).

### Brevilin A Blocks Cytokine Induced STATs Signaling

**Figure 5:**
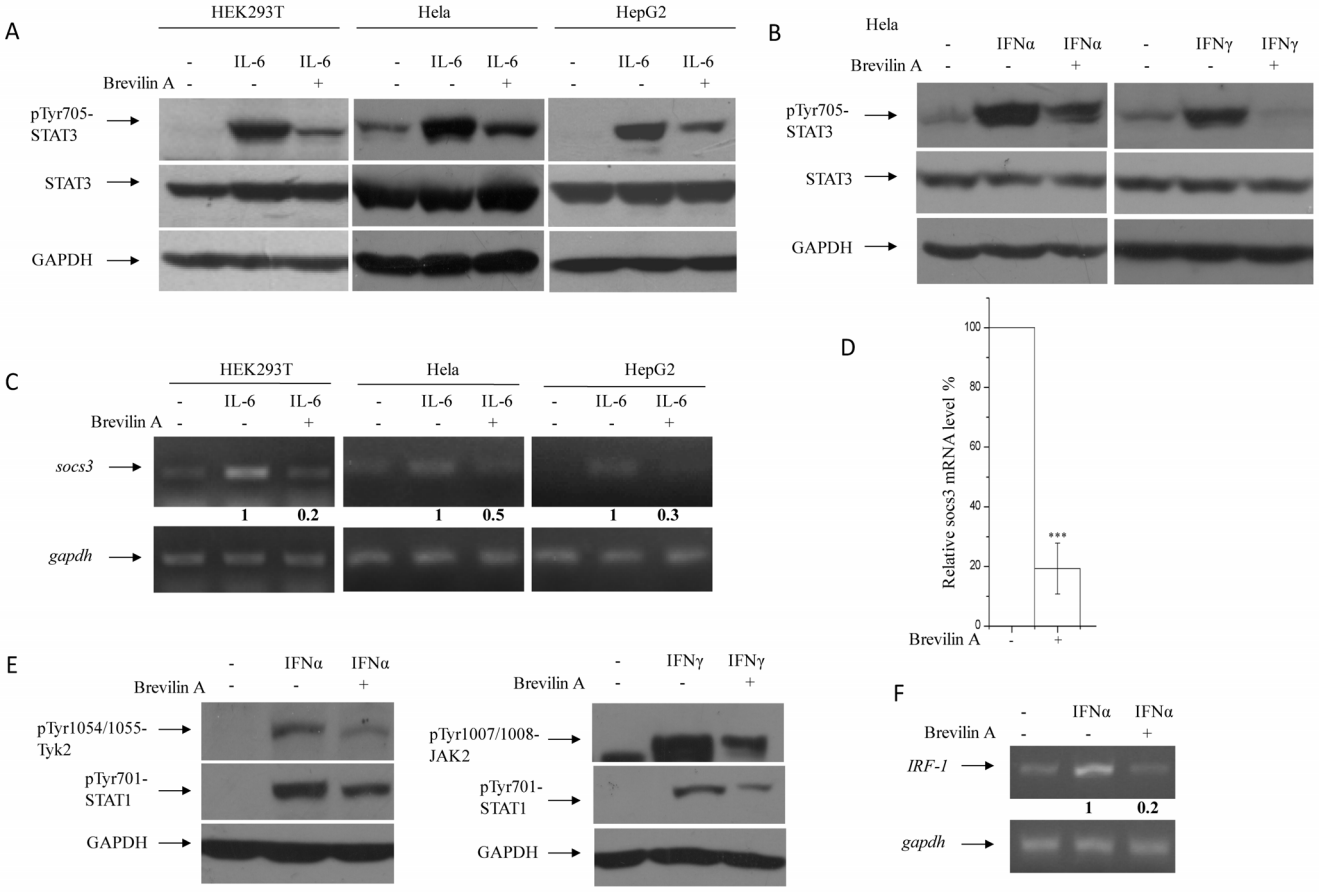
Brevilin A blocks STATs activity upon cytokine treatments. Cells were plated and cultured in 100-mm dishes for 12 h, then media were removed and replaced with fresh media without serum for another 12 h. Brevilin A (10 µM) was added for 30 min pretreatment, then cells were treated with IL-6 (250 ng/ml), IFNα (5000 U/ml) and IFNγ (1500 U/ml) for 2 h. STAT3 phosphorylation were then analyzed by Western-Blot (A and B). (C) Cells were plated and cultured in 100-mm dishes for 12 h, then media was removed and replaced with fresh media without serum for another 12 h. Brevilin A was added for 30 min pretreatment (HEK293T, 10 µM; Hela and HepG2, 15 µM ), then cells were treated with IL-6 (250 ng/ml), IFNα (5000 U/ml) and IFNγ (1500 U/ml) for 4 h. *Socs3* mRNA were analyzed by RT-PCR. (D) Real-time qPCR analysis of *socs3* mRNA in HEK293T cells treated with Brevilin A in the presence of IL-6 (250 ng/ml). (E) Brevilin A inhibits IFNα and IFNγ induced Tyk2 and JAK2 phosphorylation respectively, as well as STAT1 tyrosine phosphorylation in Hela cells as treated in (B). (F) *IRF* mRNA were analyzed by RT-PCR in Hela cells in the presence of IFNα as treatments described above.

Cytokines, like interleukins and interferons, usually induce STAT3 activation through the canonical JAK-STAT pathway. It has been reported that STAT3 was activated in DU145 and MDA-MB-468 through IL-6 autocrine loops [34], [35]. Here, in the presence of additional IL-6 treatment, we found that Brevilin A could inhibit STAT3 activation in response to IL-6 induction in HEK293T, Hela and HepG2 cells (Fig. 5A). To test whether this inhibition by Brevilin A was involved in other cytokines mediated STAT3 activation, IFNγ and IFNα were used. Briefly, IL-6 induced STAT3 activation through the IL6R-gp130-JAK pathway [36], while IFNγ and IFNα induced it by activating Type II- and Type I- interferon receptor-JAK pathway respectively [37]. After pretreatment of Hela with Brevilin A, Tyr705 phosphorylation of STAT3 was greatly inhibited as expected (Fig. 5B). Transcription of *socs3* (suppressor of cytokine signaling protein 3) gene is regulated by STAT3 activation directly in response to cytokines like IL-6 [38], so the mRNA level of *socs3* usually reflects the transcriptional activity of STAT3. We measured the mRNA level of *socs3* in response to IL-6 with or without Brevilin A pretreatment by RT-PCR in HEK293T, Hela and HepG2 cells. Brevilin A inhibited STAT3 mediated *socs3* transcription in all these cells dramatically (Fig. 5C). Real-time PCR results showed approximate 70% reduction of *socs3* mRNA after treated with Brevilin A in the presence of IL-6 in HEK293T cells (Fig. 5D).

### Brevilin A Blocks Janus Kinase Activity

**Figure 6:**
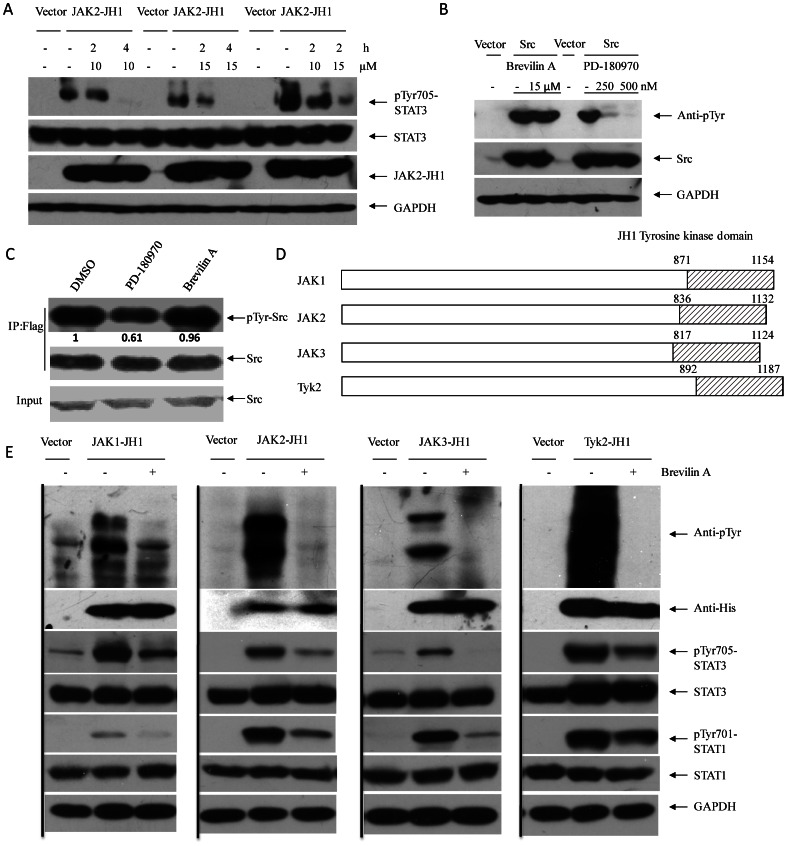
Brevilin A blocks JAKs-JH1 tyrosine kinase domain over-expression induced protein phosphorylation. (A) HEK293T cells expressing JAK2-JH1 were treated with Brevilin A (10 µM and 15 µM) for 2 h and 4 h. STAT3 tyrosine 705 phosphorylation was then detected. (B) HEK293T cells over-expressing c-Src were treated with Brevilin A (15 µM) or PD-180970 (250 nM and 500 nM) for 4 h. Then c-Src induced tyrosine phosphorylation was detected with anti-phosphotyrosine antibody. (C) HEK293T cells over-expressing Flag-tagged c-Src were treated with DMSO, Brevilin A (15 µM) and PD-180970 (500 nM) for 4 h separately. Then c-Src was immuno-precipitated with anti-Flag beads for Western-Blot analysis. (D) JH1 domain of JAK1, JAK2, JAK3 and Tyk2. (E) HEK293T cells expressing JAKs-JH1 were treated with Brevilin A (15 µM) for 4 h. JH1 induced total tyrosine phosphorylation, STAT3 and STAT1 phosphorylation were detected with indicated antibodies. Before treatment (A, B, C and E), cells were plated and cultured in 100-mm dishes for 12 h, and then media were removed and replaced with fresh media without serum for another 12 h.

**Figure 7:**
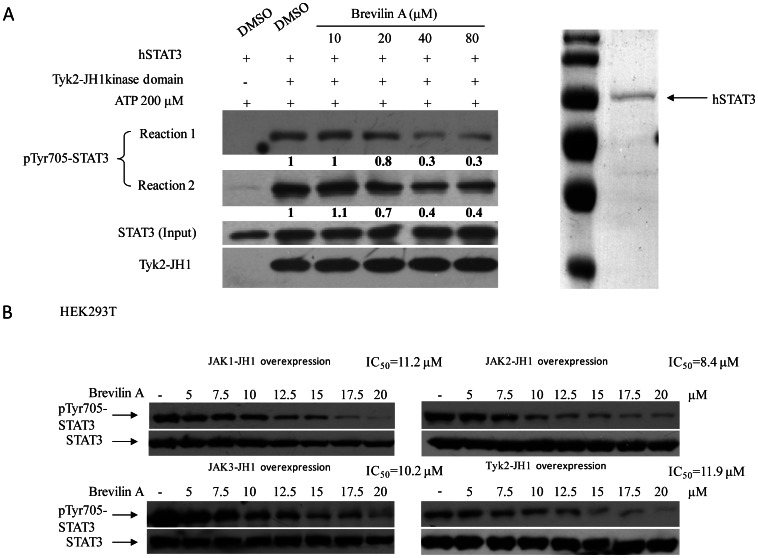
Brevilin A blocks JAKs-JH1 tyrosine kinase activity *in vitro*. (A) Purified hSTAT3 protein and immuno-precipitated Tyk2-JH1 kinase were used for kinase assay, in the presence of Brevilin A concentration series at 10, 20, 40, and 80 µM. Western-Blot showed that Brevilin A inhibited Tyk2-JH1 kinase activity directly (A, left). The right lane in (A) was the Coomassie Brilliant Blue staining of purified hSTAT3 after SDS-PAGE. Antibody against pTyr705-STAT3 was used for detecting STAT3 phosphorylation. (B) IC_50_ measurements of four JAKs-JH1 truncated kinases. HEK293T cells expressing JAKs-JH1 were plated and cultured in 100-mm dishes for 12 h, then media was removed and replaced with fresh media without serum for another 12 h. Cells were then treated with Brevilin A at different concentrations for 4 h (5 µM, 7.5 µM, 10 µM, 12.5 µM, 15 µM, 17.5 µM and 20 µM). DMSO was used as control.

Since Brevilin A could inhibit JAK2 and Tyk2 phosphorylation in response to IFNγ and IFNα (Fig. 5E), we then tested the effects of Brevilin A on STAT1 signaling. Results indicated that STAT1 phosphorylation and its target gene *IRF1* were decreased in the presence of Brevilin A after cytokine induction (Fig. 5E and 5F). These features reveals that the potential direct inhibitory targets of Brevilin A may locate upstream of STAT3 and STAT1 signaling. It unlikely seems that Brevilin A could affect cytokine receptors or co-receptors (gp130, *e.g*.) either, according to results that different cytokine-receptor mediated activation was inhibited in several different treatments (Fig. 5A and 5B). Then we focused on activities of JAK members. Each JAKs family member contains seven conserved domains, named Janus homology domains 1 to 7 (JH1-7), of which the JH1 domain is the tyrosine kinase domain and usually exhibits constitutive enzymatic activity [39], [40]. JAK2-JH1 domain encoding from 836-1132 aa (Fig. 6D) was cloned into plv-SV40-puro lentivirus expression vector [27]. HEK293T cells were then infected with virus and selected for stable pools over-expressing JAK2-JH1 domain. STAT3 Tyr705 phosphorylation was induced in this transduced cell pools and Brevilin A exhibited significant inhibition on this over-expression induced phosphorylation (Fig. 6A), indicating that Brevilin A could block JAK2-JH1 tyrosine kinase activity. The Src kinase has also been proved to be one of major activator of STAT3 which catalyzes Tyr705 phosphorylation in some cancer cells [41]. To investigate whether Brevilin A inhibits Src induced catalysis, c-Src was over-expressed in HEK293T cells. Importantly, Brevilin A does not block Src over-expression-induced phosphorylation of total cell extracts (main phosphorylated substrate) by comparing with a known Src inhibitor, PD-180970 (Fig. 6B). Then c-Src transfected HEK293T cells were pretreated with DMSO, PD180970 and Brevilin A for 4 hours, and Src protein was immunoprecipitated for further analysis. IP results showed that PD180970 was able to decrease Src phosphorylation while Brevilin A was not (Fig. 6C). To investigate whether the other three members of JAKs family were involved in Brevilin A mediated phosphorylation inhibition, HEK293T cells were over-expressed with JAK1-JH1, JAK3-JH1 or Tyk2-JH1. Figure 6D represents the regions of JAKs-JH1 domains over-expressed in HEK293T cells. All four types of JAKs-JH1 over-expressions could induce tyrosine phosphorylation of total substrates, including STAT3 and STAT1 phosphorylation. Brevilin A treatment again attenuated this phosphorylation remarkably (Fig. 6E). To verify whether Brevilin A was able to inhibit JAKs-JH kinase domain directly, Tyk2 was chosen for further *in vitro* kinase assay. We precipitated Tyk2-JH1 kinase domain and incubated it with recombinant hSTAT3 protein at different doses of Brevilin A. As expected, Brevilin A could inhibit STAT3 phosphorylation catalyzed by Tyk2-JH1 kinase domain *in vitro* (Fig. 7A). Based on this direct effect, IC_50_s could be measured by evaluating STAT3 tyrosine phosphorylation changes in JAKs-JH1 kinase domain over-expressed HEK293T cells (Fig. 7B). The values of four IC_50_s didn’t show much difference, and corresponded closely to the value got by luciferase assay as shown in Fig. 2C.

## Discussion

High-throughput drug screening for specific inhibitors based on stable constitutive activated signals has been considered a more effective way than classical ways which require additional signal stimulation before screening. Our A549R screening cell line also follows this effective principle and shows high stability even after more than 20 continuous passages. Therefore, with this stable cell line and its corresponding standard operating procedure, screening for inhibitors involved in STAT3 signaling become easier.

Persistent STAT3 activity as described previously may contribute to many cancer progressions, most of which show JAKs, Src or Receptor Tyrosine Kinase abnormalities. Here, with a screening system based on luciferase reporter in A549 cells, we finally identified a natural product Brevilin A as a JAKs inhibitor by inhibiting JAKs-JH1 kinase domain. Super activation of JAK family was usually observed in hematologic diseases. Some JAK mutations were found in high-risk childhood acute lymphoblastic leukemia (ALL) [42]. Single mutation of JAK2 V617F,which represented constitutive tyrosine kinase activation, was associated with myeloproliferative disorders [43]–[46]. JAK1 and JAK3 mutations were also found in human acute leukemias and solid cancers [47], [48]. Some human autoimmune diseases, like rheumatoid arthritis, are sensitive to JAK inhibitors. Thus these specific inhibitors involved in JAK-STAT signal pathway could act as potential effective drugs in rheumatoid arthritis and other related diseases [49]. In our investigations, Brevilin A represented higher degree of signal inhibition than direct cytotoxicity by comparing its effects on a A549R model cell line, as well as effects among normal hTERT-BJ, JAK-STAT signal dependent DU145 and MDA-MB-468 cells. Those tumor cells (like Hela and HepG2 in our investigations, data not shown), of which the growth is less dependent on JAK-STAT signals, then showed lower growth inhibition by Brevilin A.

Of the main targets of over-activated JAKs, STAT3 (sometimes STAT5 as well) is most concerned due to its novel roles in cancers. JAK inhibitors will work perfectly to inhibit STAT3 phosphorylation in these diseases. Brevilin A showed high specificity on Janus Kinase activity and following STAT3 signaling without directly affecting some other signals, including p65, AKT and GSK-3β phosphorylation, as well as Src kinase activity. Although it appeared sometimes in our investigations that STAT3 phosphorylation could be affected by Brevilin A in serum-starved Src over-expressing HEK293T cells (not shown), the most significant induction, as well as Src phosphorylation itself shown in Fig. 6B and Fig. 6C didn’t change after Brevilin A treatment, while Src inhibitor PD-180970 blocked Src phosphorylation dramatically, revealing that Brevilin A does not suppress Src activity directly. We suppose this ambiguous inhibition of STAT3 might be due to a secondary effect of Brevilin A on JAKs (no direct inhibition of Src catalysis to STAT3) in Src over-expressing cells, since it seemed that both JAK2 and Tyk2 were activated in Src-transformed human cells [50], which were also observed in our experiments. However,although we have examined a number of signaling cascades, including p65, AKT, GSK-3β and Src, which were not affected significantly by Brevilin A at the concentrations/time we evaluated, given the limited number of kinases/pathways we examined, additional studies would be necessary to determine whether Brevilin A might inhibit other kinases or pathways beyond the JAKs for a better understanding of this compound.

Brevilin A, as a small molecular from natural products, although has been reported to be involved in the rescue of multidrug resistance by down-regulating MDR1 expression (*Changlong Li*, doctoral thesis in Chinese, 2008) [51], the mechanistic details is actually unknown. It has been recently reported that STAT3 inhibition reversed drug resistance of leukemia cells by down-regulating MDR1 [52]. Our data presented here indicates that the roles of Brevilin A in JAKs inhibition may be able to reverse this drug resistance in their MDR models. Therefore, Brevilin A can be used in combination treatments with other chemotherapeutics for a better prognosis.

## Supporting Information

Figure S1 **Map of SIE-luc-puro vector derived from pGL4.20.** A sequence containing 16× SIE plus with one TATA box was inserted into pGL4.20 between KpnI and HindIII. The HindIII site was eliminated during DNA ligation. Puromycin-resistant gene is driven by SV40 promoter.
(TIF)

Figure S2 **Flow chart and summary of drug screening.**
(TIF)

Table S1 **Library information of 1440 compounds from BioBioPha Co., Ltd.**
(XLS)

Table S2 **First round screening results with fluorescence ratio between treated and control wells.**
(XLS)

Table S3 **Luciferase and MTT assays for second round screening.**
(DOC)

Table S4 **First round screening results with Z score.**
(XLS)
