# Supplementary figures and images for: Brevilin A, a Novel Natural Product, Inhibits Janus Kinase Activity and Blocks STAT3 Signaling in Cancer Cells
Source: PLoS One. 2013 May 21;8(5):e63697. doi: 10.1371/journal.pone.0063697 (PMC3660600; doi:10.1371/journal.pone.0063697)

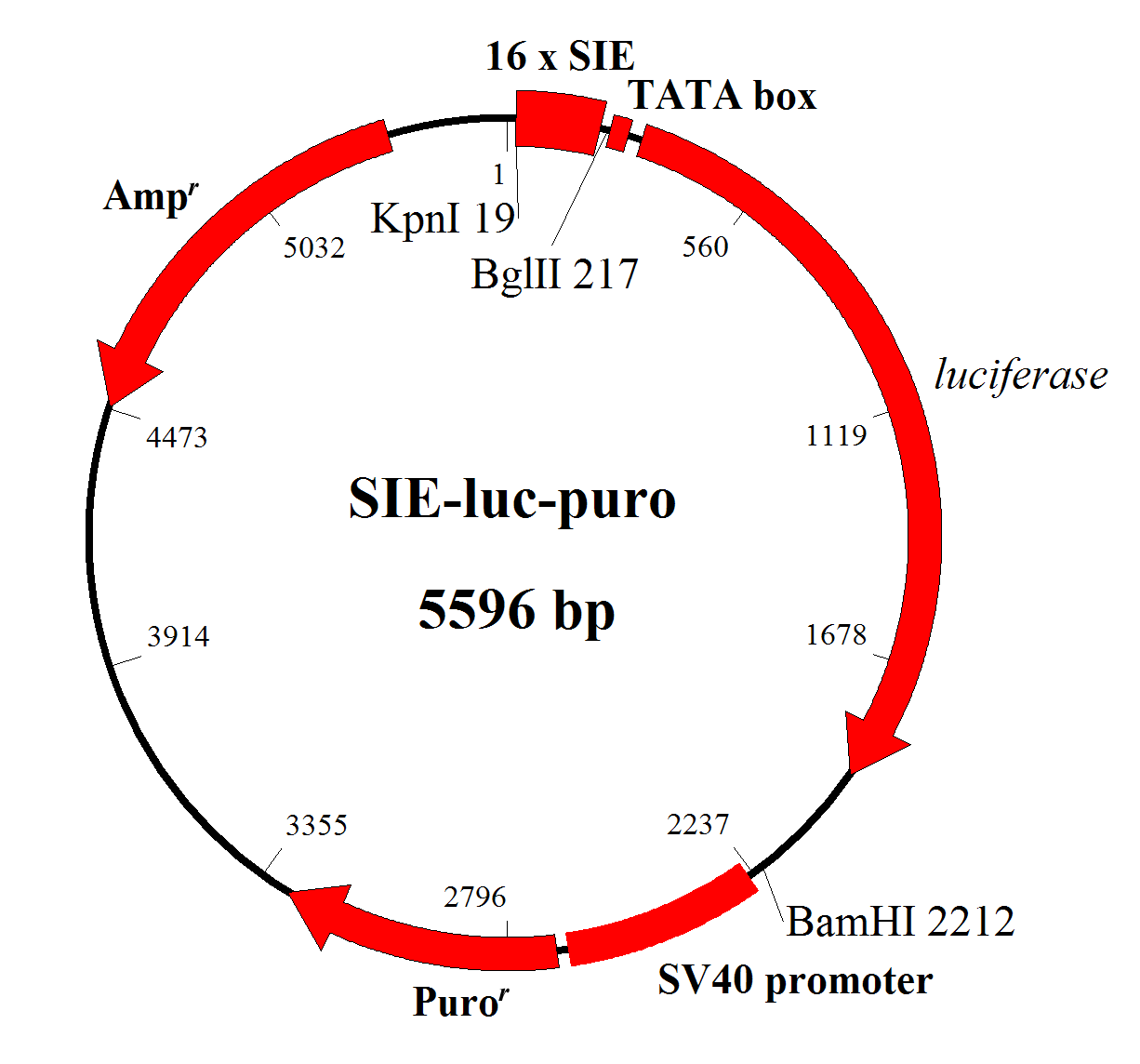

Supplement: Figure S1 — Map of SIE-luc-puro vector derived from pGL4.20. A sequence containing 16× SIE plus with one TATA box was inserted into pGL4.20 between KpnI and HindIII. The HindIII site was eliminated during DNA ligation. Puromycin-resistant gene is driven by SV40 promoter. (TIF) [file pone.0063697.s001.tif]

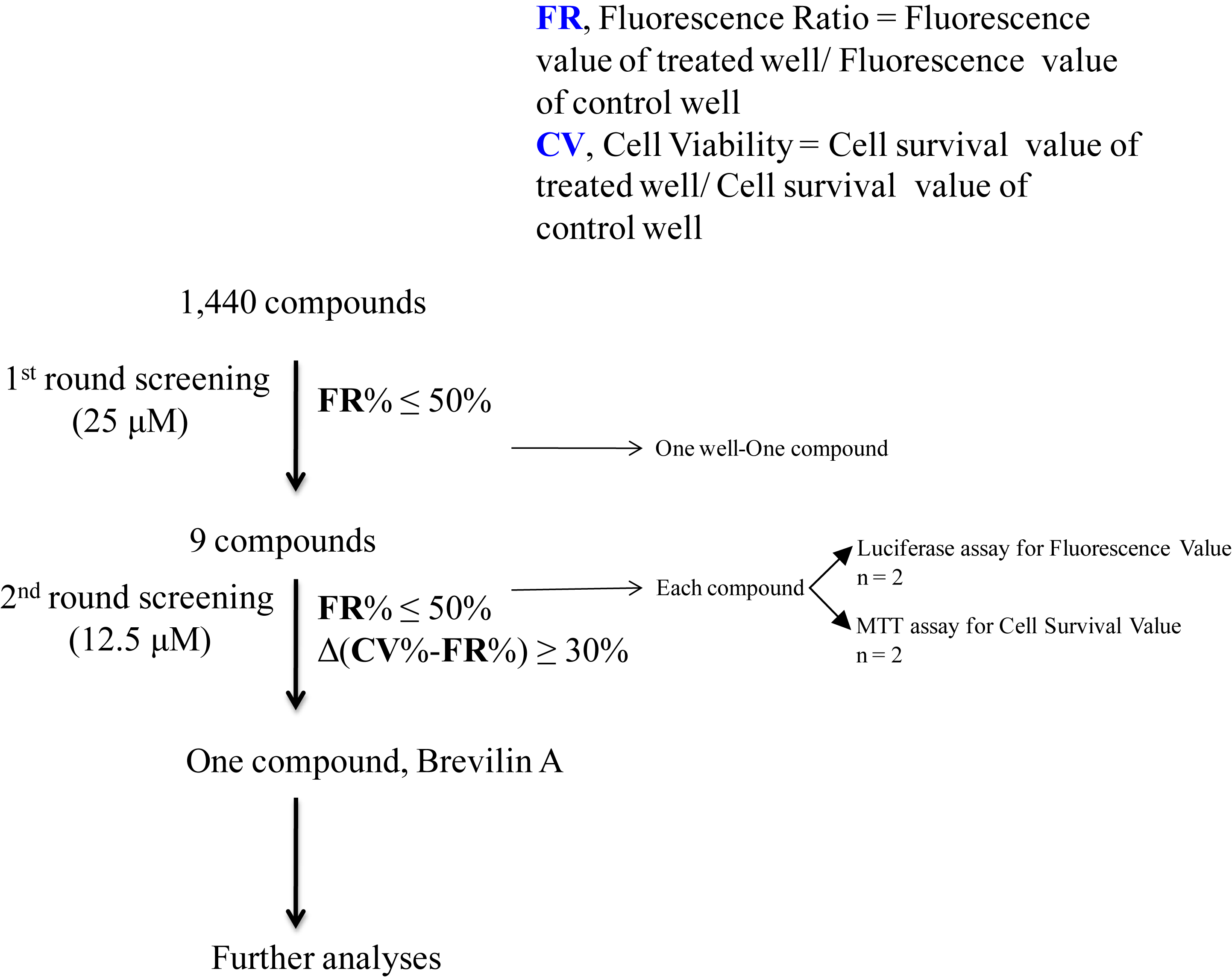

Supplement: Figure S2 — Flow chart and summary of drug screening. (TIF) [file pone.0063697.s002.tif]
