# Supplementary material for: Brevilin A, a Novel Natural Product, Inhibits Janus Kinase Activity and Blocks STAT3 Signaling in Cancer Cells
Source: PLoS One. 2013 May 21;8(5):e63697. doi: 10.1371/journal.pone.0063697 (PMC3660600; doi:10.1371/journal.pone.0063697)
Supplement: Table S3 — Luciferase and MTT assays for second round screening. (DOC) [file pone.0063697.s005.doc]

Table S3: Luciferase and MTT assays for second round screening.

| Compounds Name | Plate Location | Fluorescence ratio(Average treated value/ Average control value *100%) | Cell viability(Average treated value/ Average control value *100%) |
| --- | --- | --- | --- |
| Cudraxanthone D | 5-D10 | 61.5 | 78.6 |
| Murrangatin diacetate | 6-F3 | 107.2 | 101.6 |
| Cleomiscosin A | 8-B8 | 104.6 | 93.3 |
| Ervamycine | 11-G11 | 102.8 | 92.9 |
| Brevilin A | 12-C4 | 41.8 | 94.3 |
| Ceanothic acid acetate | 13-A8 | 94.9 | 100.8 |
| Fraxidin | 14-G6 | 93.3 | 110.1 |
| Sanguinarine | 16-F2 | 384.2 | 36.1 |
| Chonglou Saponin VII | 16-F7 | 76.6 | 100.5 |
| Positive Control | IL-6 | 292.2 | 95.2 |
| Positive Control | PD-180970 | 61.2 | 95.3 |
